# Detection of rabbit IgG by using functional magnetic particles and an enzyme-conjugated antibody with a homemade magnetic microplate

**DOI:** 10.1186/s13065-015-0088-1

**Published:** 2015-02-22

**Authors:** Hweiyan Tsai, Yi-Hsuan Lu, Huan-Xuan Liao, Shih-Wei Wu, Feng-Yih Yu, Chwan Bor Fuh

**Affiliations:** School of Medical Applied Chemistry, Chung Shan Medical University, Taichung, 402 Taiwan; School of Biomedical Sciences, Chung Shan Medical University, Taichung, 402 Taiwan; Department of Applied Chemistry, National Chi Nan University, Puli, Nantou, 545 Taiwan; Department of Medical Education, Chung Shan Medical University Hospital, Taichung, 402 Taiwan

**Keywords:** Immunoassay, Functional magnetic particles, Magnetic separator

## Abstract

**Background:**

The enzyme-linked immunosorbent assay (ELISA) has been used for diagnosing medical and plant pathologies. In addition, it is used for quality-control evaluations in various industries. The ELISA is the simplest method for obtaining excellent results; however, it is time consuming because the immunoreagents interact only on the contact surfaces. Antibody-labeled magnetic particles can be dispersed in a solution to yield a pseudohomogeneous reaction with antigens which improved the efficiency of immunoreaction, and can be easily separated from the unreactive substances by applying a magnetic force. We used a homemade magnetic microplate, functional magnetic particles (MPs) and enzyme-labeled secondary antibody to perform the sandwich ELISA successfully.

**Results:**

Using antibody-labeled MPs enabled reducing the analysis time to one-third of that required in using a conventional ELISA. The secondary antibody conjugated with horseradish peroxidase (HRP) was affinity-bound to the analyte (IgG in this study). The calibration curve was established according to the measured absorbance of the 3, 3′, 5, 5′-tetramethybezidine–HRP reaction products versus the concentrations of standard IgG. The linear range of IgG detection was 114 ng/mL–3.5 ng/mL. The limit of detection (LOD) of IgG was 3.4 ng/mL. The recovery and coefficient of variation were 100% (±7%) and 116% (±4%) for the spiked concentrations of 56.8 ng/mL and 14.2 ng/mL, respectively.

**Conclusion:**

Pseudohomogeneous reactions can be performed using functional MPs and a magnetic microplate. Using antibody-labeled MPs, the analysis time can be reduced to one-third of that required in using a conventional ELISA. The substrate–enzyme reaction products can be easily transferred to another microplate, and their absorbance can be measured without interference by light scattering caused by magnetic microbeads. This method demonstrates great potential for detecting other biomarkers and in biochemical applications.

**Graphical Abstract:**
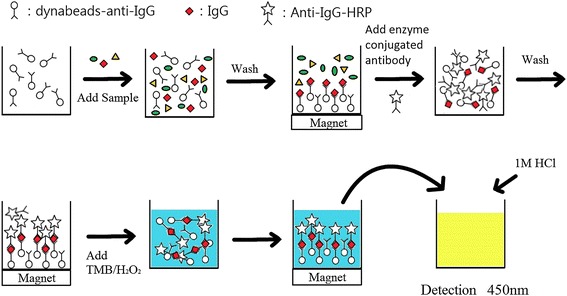
A magnetic ELISA with convenient magnetic microplate.

**Electronic supplementary material:**

The online version of this article (doi:10.1186/s13065-015-0088-1) contains supplementary material, which is available to authorized users.

## Background

In recent years, functionalized magnetic particles have attracted considerable research interest for many biological applications such as biomedicine [1-3], isolation of specific DNA [4], and manipulation of cells [5,6]. In addition, MPs have been used for detecting biomarkers by using superconducting quantum interference device (SQUID) [7-9] and magnetic resonance [10]. We used MPs for detecting biomarkers by using various techniques [11-17], such as (1) predeposited the functionalized MPs in the thin channels coupling with a magnet, then captured the biomarker antibodies and analyzed by counting particles or off-line measurement of fluorescent intensity. (2) detected biomarker antibodies using functional nanomagnetic and fluorescent nanoparticles in magnetic microplate.

A sandwich enzyme-linked immunosorbent assay (ELISA) is a frequently employed bioanalytical assay that involves using an antibody-labeled solid phase to detect the presence of a substance, generally an antigen, in a liquid sample. An enzyme-conjugated secondary antibody is then added to form a sandwich structure. Thus, the enzyme-catalyzed substrate reaction increases the sensitivity of the immunoassay. The ELISA has been used for diagnosing medical and plant pathologies. In addition, it has been used for quality-control evaluations in various industries. The ELISA is the simplest method for obtaining excellent results; however, it is time consuming because the immunoreagents interact only on the contact surfaces. We have improved the efficiency of the antigen–antibody reaction by integrating the sandwich immunoassay using functional magnetic and fluorescent nanoparticles in magnetic microplate. The magnetic microparticles were not suitable for direct measurement by microplate reader due the light scatting of the microparticles. In this study, we attempted to overcome this limitation using functional magnetic microparticles and an enzyme-conjugated antibody in a magnetic microplate. This method has many potential advantages which were reported in previous studies [17], such as (1) the amount of proteins immobilized on the particles is consistent in the same batch, which can be used for performing several reactions. (2) The magnetic microparticles (MPs) with avidin, carboxyl, or amino functional groups are commercial available which made antibody labeling easily. (3) MPs can be dispersed in a solution to yield a pseudohomogeneous reaction with antigens and can be easily separated from the unreactive substances by applying a magnetic force. (4) They can be redispersed in the solution after removing the magnetic force. (5) The enzyme-conjugated antibody can react with substrates pseudohomogeneously, and the products can be easily transferred from one microplate to another. The absorbance of the products can be measured without interference by light scattering caused by magnetic microbeads. The homogenous immunoreactions are more efficient than that of reaction on the surface of microplates [18]. Thus, in using antibody-labeled MPs, the time required for analysis is expected to be less than that required by a conventional ELISA. Most literatures on magnetic particle-based ELISA were processed in tubes [19,20]. The washing steps were done with one tube by one tube or with commercial magnetic separators, such as fully automated multisampling separators. The automated multisampling separators are expensive. Thus, we fabricated a practical and inexpensive magnetic microplate. The important contribution of our current work is the integration of microplate ELISA with homemade magnetic microplate. The process of the microplate ELISA will be more easily adopted in the clinical laboratory than tube-ELISA and the home made magnetic microplate is inexpensive.

Rabbits are among the most commonly used experimental animals in the areas of biochemical research and medical products. The serum immunoglobulin levels of an animal reflect its immune status. One of our coworkers used rabbits for performing immune experiments. Therefore, we used rabbit IgG as a model analyte to demonstrate our detection method. In this study, MPs were labeled with anti-IgG, then IgG from the sample was bound to anti-IgG-MPs. A secondary antibody conjugated with horseradish peroxidase (HRP) was then used to bind to IgG. In the final step, enzyme substrates were added. The subsequent reaction produced a color change, and the absorbance of the product was measured.

## Materials and methods

### Chemicals and materials

An affinity isolated antibody, a buffered aqueous solution of biotinlyated antirabbit IgG antibody (whole-molecule), was produced in goat; rabbit IgG purified from a normal rabbit serum by using fractionation and ion-exchange chromatography; phosphate-buffered saline (PBS); dimethyl sulfoxide (DMSO); and 3,3′,5,5′-tetramethybezidine (TMB) were purchased from Sigma-Aldrich (Saint Louis, MO, USA). Dynabeads® MyOne™ Streptavidin T1 (streptavidin-coupled superparamagnetic beads 1 μm in diameter) and Novex® HRP-conjugated goat antirabbit IgG (H & L) antibody were purchased from Life Technologies (Grand Island, NY, USA). Triton X-100 was purchased from Tedia (Fairfield, OH, USA). Hydrogen peroxide (H_2_O_2_, 35%) was purchased from Shimakyu’s Pure Chemicals (Osaka, Japan).

### Magnetic separator–magnetic microplate

Permanent magnets 6 mm in diameter and 13 mm in length were fixed in the wells of a microplate, and the assembled magnetic microplate (Additional file 1: Figure S1) was then placed under another microplate to form a magnetic separator. The magnetic field strength of these magnets was 4.1 (±0.2) kG at the top of separator.

### Instrumentation

A spectrometer (Flexstation 3 multimode plate reader, Molecular Devices, Sunnyvale, CA, USA) was used to measure the optical intensity.

### Functional magnetic particle preparation (anti-IgG-labeled MPs)

Streptavidin-coupled dynabeads were conjugated with biotinylated anti-IgG, based on the extremely high binding affinity of the streptavidin–biotin interaction (K_d_ = 10^−15^), and further used for developing the pseudohomogeneous immunoassay. An aliquot of 100 μL of biotinylated anti-IgG (3.3 mg/mL) was added to a centrifuge tube containing 10 mL of PBS and 10 mg dynabeads, and the tube was then gently rotated using a MACSmix™ tube rotator for 2 h at 4°C. Anti-IgG-labeled MPs were attracted by the magnets, and the MPs were washed three times with PBS to remove unreacted anti-IgG. Finally, anti-IgG-labeled MPs were reconstituted with 10 mL of PBS and divided into aliquots of 1 mL each. The aliquots were maintained at 4°C until use. The suspensions of the unreacted and washed PBS were mixed, and the protein concentration was evaluated using a Bradford reagent. The amount of labeled anti-IgG was approximately 18 μg/mg of dynabeads, which was semiquantitative based on the added amount subtracted from the amount left in the suspension. This result was consistent with that claimed by the supplier (biotinylated IgG up to 20 μg/mg of dynabeads).

### Procedures for the magnetic sandwich immunoassay

**Figure 1:**
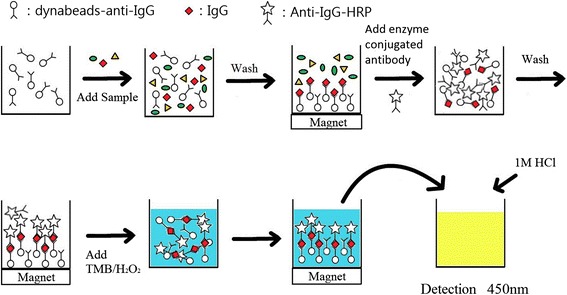
**Schematic of immunoassay procedures for a magnetic ELISA.**

Figure 1 shows the schematic of the reaction steps involved in IgG detection. This is a sandwich-type detection conducted by applying magnetic force and colorimetric detection. The procedures are briefly described as follows: In Step 1, an aliquot of 220 μL of IgG standards or serum samples, and 10 μL of 1 mg/mL anti-IgG-labeled MPs were added to the wells of the microplate. The mixture was then pipetted several times for mixing and incubated for 20 min at room temperature. In Step 2, the microplate was placed on top of a homemade magnetic microplate that attracted MPs to the bottom of the plate, and the remaining solution was gently removed. The microplates were then washed twice with PBS containing 0.1% BSA. In Step 3, 20 μL of anti-IgG-HRP (dilution ratio of 1:1000) and 200 μL of PBS were added to the microplates. The mixture was then pipetted several times for mixing and incubated for 30 min at room temperature. In Step 4, the unreacted anti-IgG-HRP was removed by washing the microplates twice with PBS containing 0.1% triton. The sandwich particles were resuspended in 200 μL of buffer solutions containing the enzyme substrate (TMB/H_2_O_2_). The enzyme substrates were 0.5 mL of TMB (2.0 mg/mL in DMSO) and 32 μL of 0.75% H_2_O_2_ freshly mixed with 10 mL of PBS. This is the reported optimized concentration of a TMB substrate [21]. In Step 5, finally, the microplate was placed on top of the homemade magnetic microplate, and 150 μL of the solution was transferred to another microplate. An aliquot of 50 μL of 1 M HCl was added to each well, and the absorbance for each sample was measured at 450 nm. All experiments were performed in triplicate. Notably, the solution of dynabeads mixed with 1 M HCl gradually turned yellow, and the beads caused light scattering. Therefore, we transferred the products of the enzyme–substrate reaction to another microplate for subsequent measurements.

A calibration curve of the absorbance was established by plotting the measured absorbance versus the various known concentrations of IgG.

### Preparation of serum and spiked samples

We drew blood from rabbits’ ears. The rabbit antisera was precipitated using (NH_4_)_2_SO_4_ to a final concentration of 50% and 35% in sequence by 100% saturated (NH_4_)_2_SO_4_ solution. The precipitate was redissolved in distilled water equal to half of the original volume and then dialyzed against 2 L PBS for 72 h at 4°C with two changes of buffer. Finally, 0.01 M PBS was added to the original volume. The rabbits were New Zealand white rabbits (body weight, 3.5 kg), which were obtained from Deer-Ho farm (Taichung, Taiwan) for immune experiments conducted by our coworkers. The animal experiments in our study were approved by the Institutional Animal Care and Use Committee at the Chung Shan Medical University (approval no. 1269). Prior to using the ELISA, the serum was diluted 10,000 times using PBS. To demonstrate the practicality of the proposed magnetic ELISA, IgG concentration in spiked serum samples was measured.

## Results and discussion

### Optimization of immunoreaction time

**Figure 2:**
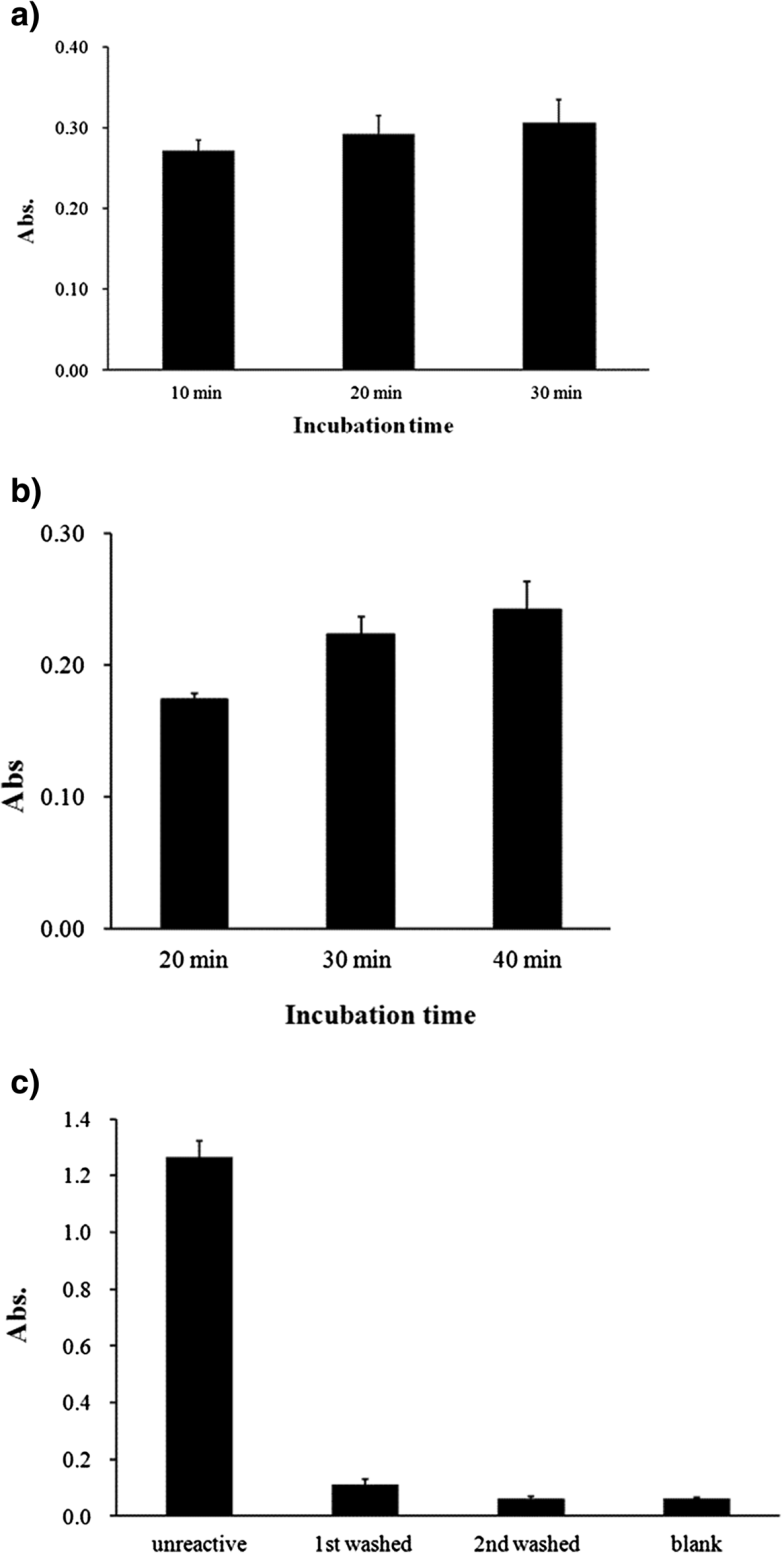
**Optimization of immunoreaction time. (a)** Effect of reaction time of anti-IgG-labeled MPs and IgG. **(b)** Effect of reaction time of anti-IgG-HRP with the microparticles to form the sandwich. **(c)** Relationship between washing times and the cleanliness of unreacted anti-IgG-HRP in the solution. “Unreactive” represents aliquots from the suspension of the mixture of anti-IgG-HRP and MP-anti-IgG-IgG after incubation. “1st washed” represents aliquots from the solution of the first washing. “2nd washed” represents aliquots from the solution after the second washing. “Blank” represents aliquots that contained only TMB/H_2_O_2_ in PBS.

The incubation time of an antibody and antigen is one of the crucial parameters for achieving a satisfactory sensitivity of an immunoassay [17]. Therefore, we varied the immunoreaction time from 10 to 30 min. Figure 2a shows the performance results as a function of the incubation time of anti-IgG-labeled MPs and IgG. The duration of the sandwich immunoreaction (IgG and anti-IgG-HRP) was 30 min. The amount of the HRP–TMB reaction products increased as the reaction time increased. We preliminarily applied 30 min for the enzyme–substrate reaction based on the balance of time consumption and detection sensitivity. The results showed that the intensity increased with longer incubation time; however, no significant difference was observed between the results of reactions performed for 20 min and 30 min (t-test, p = 0.90). Therefore, we applied an incubation time of 20 min for the first antibody–antigen reaction, and then further studied the secondary antibody reaction time of the sandwich immunoreaction (IgG and anti-IgG-HRP). Figure 2b shows the performance results as a function of the incubation time of anti-IgG-HRP and MP-anti-IgG-IgG. No significant differences were observed between the results of reactions performed for 30 min and 40 min (t-test, p = 0.33). Therefore, we set 20 min for the primary immunoreaction and 30 min for the secondary immunoreaction as the optimized immunoreaction time.

Figure 2c shows the effect of wash times on the cleanliness of the unreactive anti-IgG-HRP. We transferred the suspension of the secondary immunoreaction and washed buffers to another microplate, and then removed 20 μL of each solution to react with 200 μL of PBS containing TMB/H_2_O_2_. The results from the twice-washed buffer were not different from those of the blank that contained only the TMB/H_2_O_2_ in buffer. Therefore, we washed the microplate only twice after each immunoreaction step.

### Effect of amount of magnetic beads

**Figure 3:**
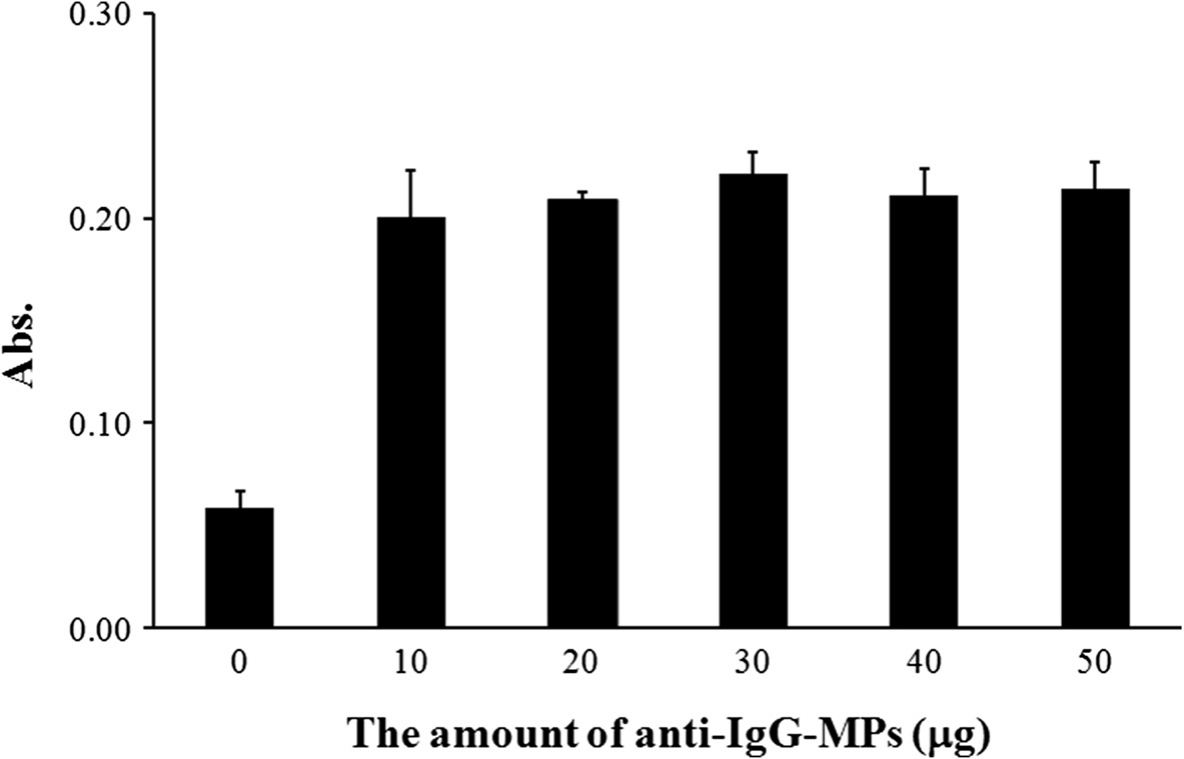
**Effect of the amount of magnetic beads on the sensitivity.** Here, 22.7 ng/mL (ie. 0.005 μg/well) of IgG with varied amounts of anti-IgG-labeled MPs were incubated for 20 min and washed twice with PBS containing 0.1% BSA. IgG was then reacted with anti-IgG-HRP for 30 min and washed twice with PBS containing 0.1% triton. Furthermore, 200 μL of PBS containing TMB/H2O2 was added, and 150-μL aliquots were transferred to another microplate after 30 min. Finally, 50 μL of 1 M HCl was added to this microplate, and its absorbance was measured at 450 nm.

The amount of dynabeads was relative to the amount of available anti-IgG; more antibodies reacted with more antigens, thereby leading to enhanced sensitivity and a wider dynamic range. By contrast, a large amount of the dynabeads caused particle aggregation. Therefore, we studied the effect of the amount of magnetic beads on sensitivity and linearity. The analysis of variance (ANOVA) statistical results (p = 0.487) showed no significant difference in the colorimetric intensities from the immunoreaction containing varied amounts of magnetic beads, as shown in Figure 3. This could have been due to the amount of labeled anti-IgG being considerably greater than the amount of IgG in the solution.

Regarding the cost of the dynabeads and particle aggregation, we performed dose-dependent measurements of IgG with 10 μg and 20 μg of anti-IgG-labeled MPs.

### Calibration curve and reproducibility

**Figure 4:**
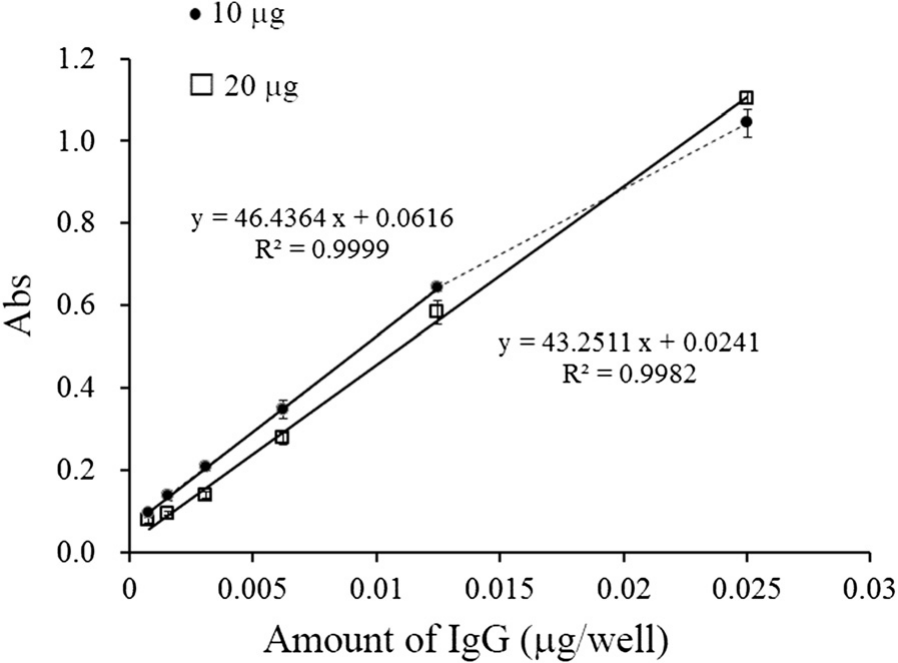
**Plots of the calibration curves of IgG.** The conditions of immunoreactions were the same as those shown in Figure 3.

Calibration curves were established using commercial rabbit IgG, which was purified from the normal rabbit serum by using fractionation and ion-exchange chromatography. The plots of colorimetric absorbance of varied concentrations of IgG for the optimal reaction time are displayed in Figure 4. The linearity, slope, LOD, and precision are as shown in Table 1. As expected, the upper concentration of the linear range from 20 μg of anti-IgG-labeled MPs was twice that from 10 μg of anti-IgG-labeled MPs. The LOD based on 3S_b_/m was 0.59 ng/mL and 3.4 ng/mL for 10 μg of anti-IgG-labeled MPs and 20 μg of anti-IgG-labeled MPs, respectively. However, no significant difference was observed between their sensitivities.

**Table 1:** **Data of analytical specifications**

**Amount of anti-IgG-MPs**	**Slope (Abs/μg)**	**R** ^**2**^	**Range (ng/mL)**	**LOD (ng/mL)**	**Precision (reproducibility)**
10 μg	46.3 (±0.3)	0.9999	56.8 ~ 3.54	0.59	2 ~ 10%
20 μg	43.3 (±0.9)	0.9982	113.6 ~ 3.54	3.4	1 ~ 9%

The importance of precision has often been emphasized for using a bead-based immunoassay in quantitative analysis. The intraassay precision of the analytical method was calculated by analyzing each concentration in triplicate per run. The relative standard deviation was 1%–5% at varied concentration levels, except for 9%–10% at 3.5 ng/mL. These results implied that the proposed method exhibited satisfactory reproducibility. The bioactivity of highly diluted HRP decayed rapidly. Aliquots of anti-IgG-HRP diluted 100 times were maintained at 4°C. The working solution of anti-IgG-HRP was adjusted to 500–1000 dilutions based on a positive control test of the anti-IgG-HRP–TMB/H_2_O_2_ reaction, which maintained an absorbance at 1.5. Thus, reproducibilities of the interassay were less than 7%.

### Determination of IgG in rabbit serum sample

An aliquot of 220 μL of dilute rabbit serum was incubated with 20 μg of anti-IgG-labeled MPs. The recovery was measured for the spiked IgG in serum at final concentrations of 56.8 ng/mL and 14.2 ng/mL. The IgG concentration in the obtained serum and spiked solutions was measured and interpreted according to the calibration curve. The IgG concentration of the rabbit serum was 7.66 mg/mL (±3%), which is consistent with that reported (5–10 mg/mL) by a previous study [22]. The recovery and coefficient of variation were 100% (±7%) and 116% (±4%) for the spiked concentrations of 56.8 ng/mL and 14.2 ng/mL, respectively.

## Conclusion

We developed an ELISA that combines the sandwich immunoassay with MPs and an enzyme-conjugated secondary antibody on a magnetic microplate for determining the IgG concentration in a buffer solution and serum. The high sensitivity of the assay was achieved using the colorimetric method for measuring the activity of the conjugated HRP. The dynamic working range was 114-3.5 ng/mL. The recovery ranged from 100% to 116%, and reproducibility ranged from 1% to 10%. In using antibody-labeled MPs, the time required for analysis was reduced to one-third of that required in using a conventional ELISA. The detection limit was 3.4 ng/mL (i.e. 2.3 × 10^−11^ M) which was lower than 10^−9^–10^−10^ M suggested by the vendors of conventional ELISA kits and time-resolved fluorescence [23]. The homemade magnetic microplate was practical and inexpensive. This method has satisfactory potential for detecting other biomarkers and in biochemical applications.

## Additional file

Additional file 1: Figure S1. The homemade magnetic microplate.
