# Supplementary figures and images for: Detection of rabbit IgG by using functional magnetic particles and an enzyme-conjugated antibody with a homemade magnetic microplate
Source: Chem Cent J. 2015 Feb 22;9:8. doi: 10.1186/s13065-015-0088-1 (PMC4350001; doi:10.1186/s13065-015-0088-1)

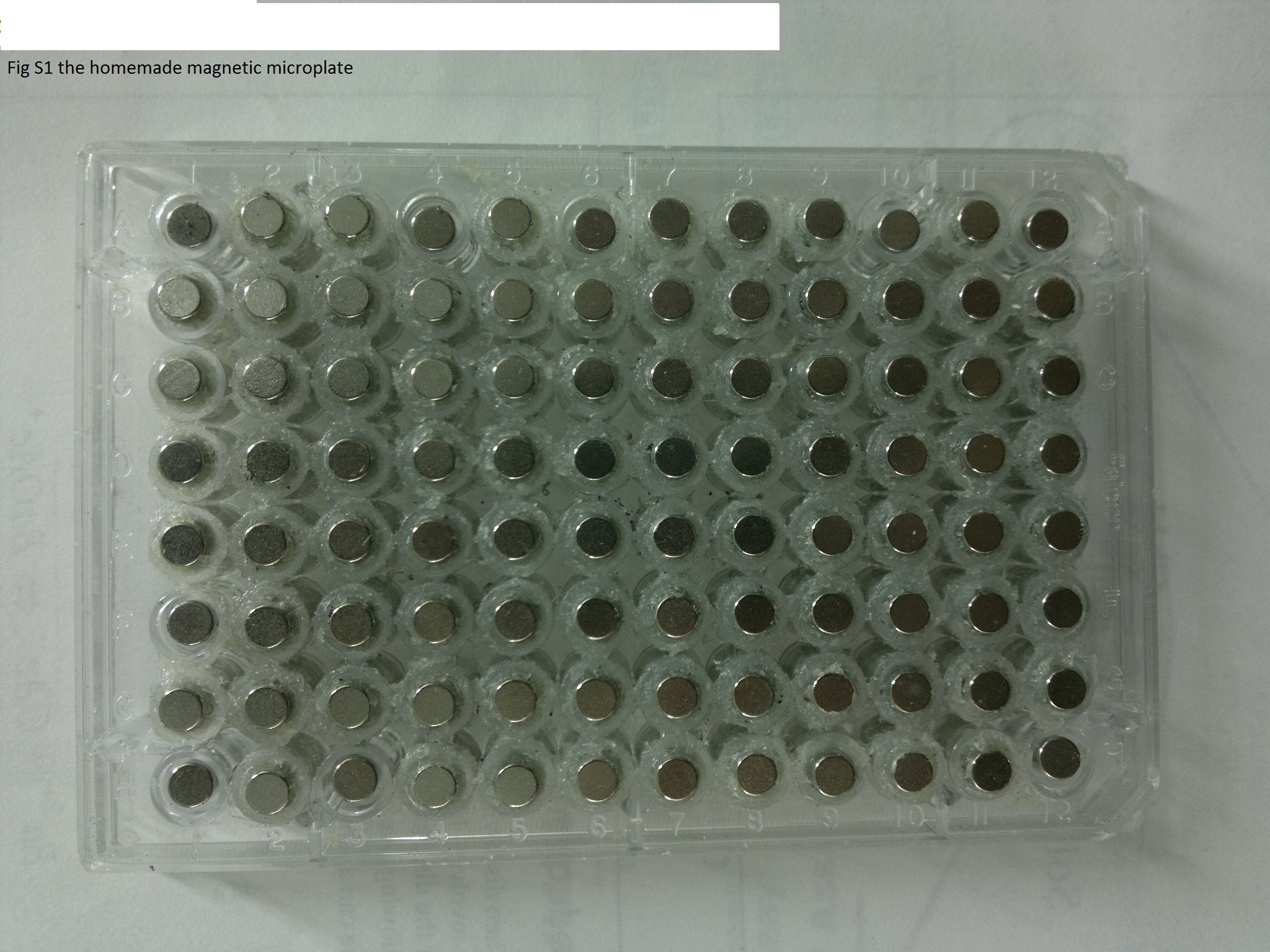

Supplement: Additional file 1: Figure S1. — The homemade magnetic microplate. [file 13065_2015_88_MOESM1_ESM.jpeg]
